# A New Approach on Estimation of Solubility and *n*-octanol/water Partition Coefficient for Organohalogen Compounds

**DOI:** 10.3390/ijms9060962

**Published:** 2008-06-02

**Authors:** Shuo Gao, Chenzhong Cao

**Affiliations:** 1 School of Chemistry and Chemical Engineering, Central South University, Changsha, 410083, P. R. China; E-mail: shuogao@yahoo.cn; 2 School of Chemistry and Chemical Engineering, Hunan University of Science and Technology, Xiangtan, 411201, P. R. China; E-mail: czcao@hnust.cn

**Keywords:** *n*-Octanol/water partition coefficient, aqueous solubility, organohalogen compounds, quantitative structure-property relationship, HOMO-LUMO interaction

## Abstract

The aqueous solubility (logW) and *n*-octanol/water partition coefficient (logP_OW_) are important properties for pharmacology, toxicology and medicinal chemistry. Based on an understanding of the dissolution process, the frontier orbital interaction model was suggested in the present paper to describe the solvent-solute interactions of organohalogen compounds and a general three-parameter model was proposed to predict the aqueous solubility and *n*-octanol/water partition coefficient for the organohalogen compounds containing nonhydrogen-binding interactions. The model has satisfactory prediction accuracy. Furthermore, every item in the model has a very explicit meaning, which should be helpful to understand the structure-solubility relationship and may be provide a new view on estimation of solubility.

## 1. Introduction

Aqueous solubility (logW) and *n*-octanol/water partition coefficient (logP_OW_) of compounds have long been recognized as the key molecular properties and are widely used in such diverse areas as pharmaceutics, biochemistry, environmental chemistry, toxicology, chemistry and chemical engineering. Drug delivery, transport, and distribution; prediction of environmental fate; and development of analytical methods depend on solubility and partition properties [1, 2]. As a consequence, it is of considerable value to have practical knowledge of the logW and logP_OW_ values for molecules. The measurement of logW or logP_OW_ through the synthesis of a compound and then its subsequent experimental determination is time-consuming and expensive. Hence, there is strong interest in the structure-based prediction of logW or logP_OW_ for rational development of new drugs and for reasonable assessment of the environmental impact of chemicals before they were released into the environment. Not surprisingly, numerous methods for the prediction of aqueous solubility or partition coefficients have been suggested in the literature [3–19]. Fortunately, Jorgensen did a very good review on the prediction methods of logW for organic compounds [20]. Recently, Kühne has made a comparison among those widely used methods and pointed out that “every method has its method-specific application domains” [21]. Thus, new methods for supplementing existing approaches are required.

It is well known that organohalogen compounds have been manufactured and used in the chemical industry as solvents, propellants, additives, cooling agents, and insecticides for many years [22]. In addition, these compounds can be formed during combustion processes in waste incineration. Generally, organohalogen compounds, such as polychlorinated biphenyls (PCBs), polybrominated biphenyls (PBBs), polychlorinated benzenes, polybrominated benzenes and polychlorinated naphthalenes (PCNs) and so on, have some extent negative impact on the environment and the ecology. Thus the assessment of the environmental risk of these compounds, which can be roughly done by studying their logW or logP_OW_, is very important. Recently, Padmanabhan [23], Lü [24], and Zou [25] proposed QSPR models to predict the logP_OW_ of PCBs, and obtained good prediction accuracy. In the present paper, based on an understanding of the processes involved in dissolution, a new and very simple method was suggested to predict the logP_OW_ and logW for some halogen containing organic compounds. The present method has a good prediction accuracy and every term in the presented equation has an explicit physical and chemical meaning.

## 2. Methodology

The dissolution of a solute in a solvent can conceptually take place in two stages: (i) a sizable hole or cavity has to be formed in the solvent phase to accommodate the solute molecule; (ii) the solute molecule is then inserted into the hole, and then interacts with the solvent molecules around it. After the above two steps, a stable solution is formed [26].

At the first stage of the dissolution, an input energy or enthalpy (E_input_) is needed to separate the solvent molecules, i.e., to overcome the solvent-solvent cohesive interactions. This energy is proportional to the size or volume of the solute molecule. The second stage of the dissolution is an exothermic process. The output energy (E_output_) in this stage for organic compounds having no (or very weak) hydrogen-binding interactions with solvent molecules, is, in our opinion, correlated with the interaction of the frontier orbitals of the solute molecules (FMO_solute_) and solvent molecules (FMO_solvent_). In other words, ignoring the interaction of the hydrogen-binding interactions resulting from solute and solvent molecules, E_output_ is mainly determined by the interactions between solvent’s HOMO (HOMO_solvent_) and solute’s LUMO (LUMO_solute_), and between solute’s HOMO (HOMO_solute_) and solvent’s LUMO (LUMO_solvent_), viz.:

(1) $${\text{E}}_{\text{output}}=b'(E_{{\text{HOMO}}_{\text{solute}}}-E_{{\text{LUMO}}_{\text{solvent}}})+c'(E_{{\text{HOMO}}_{\text{solvent}}}-E_{{\text{LUMO}}_{\text{solute}}})$$

According to the above statements, the following equation was proposed to predicted the logW for the organohalogen compounds having no (or very weak) hydrogen-binding interactions with solvent,

(2a) $$\begin{array}{c} \log W=a'V_{\text{solute}}+b'(E_{{\text{HOMO}}_{\text{solute}}}-E_{{\text{LUMO}}_{\text{solvent}}})+c'(E_{{\text{HOMO}}_{\text{solvent}}}-E_{{\text{LUMO}}_{\text{solute}}})\\ =a'V_{\text{solute}}+b'E_{{\text{HOMO}}_{\text{solute}}}-c'E_{{\text{LUMO}}_{\text{solute}}}+(c'E_{{\text{HOMO}}_{\text{solvent}}}-b'E_{{\text{LUMO}}_{\text{solvent}}}) \end{array}$$

where, *E*_*HOMO*_*solute*__, *E*_*LUMO*_*solute*__, *E*_*HOMO*_*solvent*__, and *E*_*LUMO*_*solvent*__ are the energies of the HOMOs and LUMOs of solute and solvent molecule, respectively. For a solvent of interest, e.g. water, its frontier orbitals energies are given. The last term in Eq. (2a) is an invariable, thus Eq. (2a) can be rewritten as:

(2b) $$\log W=aV+bE_{\text{HOMO}}+cE_{\text{LUMO}}+d$$

Here, *a*, *b*, *c* and *d* are the coefficients; *V*, *E*_*HOMO*_ and *E*_*LUMO*_ are the volume, the HOMO energy and the LUMO energy of the solute, respectively. The parameter *V* of a solute can be calculated by additive method, for the details one should consult Ref. [10]; The *E*_*HOMO*_ and *E*_*LUMO*_ were calculated by Gaussian 98 program (using Gaussian program packages in SYBYL 6.7 of Tripos, Inc.) at the HF/6-31G(d) level.

## 3. Regression Analysis

### 3.1. Aqueous Solubility of PCBs

**Figure 1.**
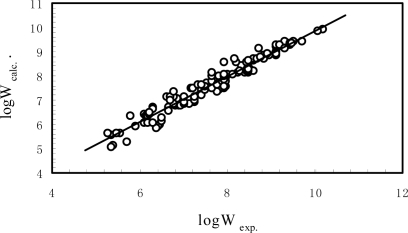
The plot of experimental aqueous solubility vs. the ones calculated by Eq. (3)

Taking some experimental logW of PCBs [11] (listed in Table 1) as the training set, we employed Eq. (2b) to carry out a regression analysis and got the following equation:

(3) $$\begin{array}{c} \log W=-0.0298V+11.1821E_{\text{HOMO}}+32.7300E_{\text{LUMO}}\\ R=0.9739,\;R^{2}=0.9485,\;S=0.26,\;n=134,\;F=804,\;R_{\text{CV}}=0.9722,S_{\text{CV}}=0.27 \end{array}$$

where, *R* is the correlation coefficient, *S* is the standard error between the experimental and estimated log*W* by Eq. (3), *n* is the number of the sample in the training set. Figure 1 is the plot of the experimental versus the calculated aqueous solubility of PCBs by Eq. (3). From the *R* and *S* value of Eq. (3) and Fig. 1, one can see that Eq. (3) has a good correlation.

The characteristics and interrelations of descriptors in Eq. (3) are given in Tables 2 and 3, respectively, which suggested that the three descriptors (*V*, *E*_*HOMO*_, and *E*_*LUMO*_) are significant descriptors and not strongly correlated with each other. According to the *t*-test values (in Table 2), the more significant descriptor appearing in Eq. (3) is the descriptor *V*, which indicated that the volume of PCB molecules is the predominant factor determining the PCB’s aqueous solubility. The *t*-score value of parameter *E*_*LUMO*_ implied that the interaction of LUMO of PCB molecule with the HOMO of water is also play a very importance role in the determination of the PCB’s aqueous solubility.

### 3.2. n-Octanol/Water Partition Coefficient of PCBs

Hantsch *et al*. [27] have indicated that there exists a linear relationship between the aqueous solubility (log*W*) and the *n*-octanol/water partition coefficient (logP_OW_) of a solute. As Eq. (2b) can express well the relationship of the structure-aqueous solubility for PCB congeners, it was also expected to be able to predict the *n*-octanol/water partition coefficient. Thus, taking some experimental logP_OW_ of PCBs [10, 23] (see Table 1) as the training set, we employed Eq. (2b) to carry out the regression analysis and got the following equation:

(4) $$\begin{array}{c} \log{\text{P}}_{\text{OW}}=0.0126V-15.6461E_{\text{HOMO}}-31.5498E_{\text{LUMO}}+0.9363\\ R=0.9610,\;R^{2}=0.9235,\;S=0.224,\;n=157,\;F=615,\;R_{\text{CV}}=0.9586,\;S_{\text{CV}}=0.23 \end{array}$$

Equation (4) has a high correlation coefficient *R* and small standard deviation *S* for predicting the *n*-octanol/water partition coefficient of PCB congeners.

## 4. Discussion

A closer analysis of the coefficients in front of the parameters in Eq. (3) can provide physical insights to understand structure-solubility relationship. The negative coefficient of the parameter *V* implied that the PCB molecule with a larger volume has a smaller log*W* value than that of the smaller PCB. That is to say, the larger PCB has lower solubility in water than that of the smaller PCB, because a larger hole has to be carved in the water layer for accepting the larger PCB molecule, which needs a larger energy input. The positive coefficient of *E*_*HOMO*_ means that the higher the HOMO of the PCB molecule, the larger the log*W* value of the PCB is. In our opinion, the higher energy of HOMO of the solute can interact with the LUMO of water more effectively, which is more energetically favorable for the formation of the solution. Thus the higher the HOMO of the PCB molecule, the more soluble it is. The higher the energy of LUMO of the solute molecule, the more effectively the LUMO of the solute molecule interact with the HOMO of water. Thus, the solubility of PCBs increases with the increase of the *E*_*LUMO*_ values.

In order to test the robustness and prediction ability of Eq. (3), a cross-validation analysis was performed. In the cross-validation analysis, a model is calculated with groups of objects (i.e., PCB congeners) omitted subsequently, followed by the prediction of the logW for the omitted objects. In the present study, Leave-One-Out (LOO) cross-validation method is employed. The internal predicted ability and the robustness of the models are characterized in terms of the corresponding leave-one-out cross-validation correlation coefficient (*R*_CV_) and the cross-validation predicted standard error (*S*_CV_), which are defined as:

(5) $$R_{CV}=\sqrt{1.0-\frac{\overset{n}{\underset{i=1}{\Sigma }}{\left(y_{i}-\hat{y_{i}}\right)}^{2}}{\overset{n}{\underset{i=1}{\Sigma }}{\left(y_{i}-\bar{y_{i}}\right)}^{2}}}$$

where *y*_*i*_ and *ŷ*_*i*_ are the experimental and predicted value, respectively. ȳ is the mean value of *y*_*i*_.

(6) $$S_{CV}=\sqrt{\frac{\overset{n}{\underset{i=1}{\Sigma }}{\left(y_{i}-{\hat{y}}_{i}\right)}^{2}}{N-M-1}}$$

where *N* is the number of samples used for model building, *M* is the number of descriptors. The *R*_CV_ and *S*_CV_ of Eq. (3) showed that Eq. (3) is robust with only 0.27 log unit for the prediction error of PCBs’ logW. The obtained parameters *R*_CV_ and *S*_CV_ also show that Eq. (4) is robust.

Recently, Padmanabhan *et al*. [23], and Lü *et al*. [24] developed the QSPR models for estimating logP_OW_ of 133 PCB congeners with prediction errors of 0.225 and 0.205 log units, respectively. In order to compare with Padmanabhan’s and Lü’s work, we employed the same data set used in their work and employed Eq.(2b) to perform the correlation analysis, namely:

(7) $$\begin{array}{c} \log{\text{P}}_{\text{OW}}=0.00538V-21.6659E_{\text{HOMO}}-41.1943E_{\text{LUMO}}+1.3603\\ R=0.9631,\;R^{2}=0.9276,\;S=0.206,\;n=133,\;F=550 \end{array}$$

The results of Eq. (4) and Eq. (7) showed that the predicted accuracy of the present model is better than that of Padmanabhan’s QSPR model and is comparable to that of Lü’s model. Examination of Eq. (4) or Eq. (7) may lead to the following significant interpretations: the value of logP_OW_ increases with the increase of *V*, which means that increase in solute size, *V*, favors wet octanol phase. The reason is that water molecule is more polar than the *n*-octanol molecule, so the cohesive energy is larger between water molecules than that between the *n*-octanol ones. Thus, more energy input is needed to create a similarly-sized hole in the more polar solvent (i.e., water phase) than that in the less polar solvent (i.e., *n*-octanol phase). Consequently, the PCB molecule tends to enter into the *n*-ocatanol phase, which is energetically more favorable. Increase the *E*_*HOMO*_ or *E*_*LUMO*_ solute favors the aqueous layer.

It is relatively easy to build a QSPR model for the congeners, while it is somewhat difficult to correlate a data set of heterogeneous compounds. Besides having a high correlation and low deviation, a valuable QSPR model should also have a large application range. Thus, in order to verify the application of Eq. (2) in more complex data sets, we combined the logP_OW_ of 157 PCBs and some other halogen substituted aromatic compounds [10, 11] (including PBBs, PCNs, and HBs, listed in Table 1) as a data set, and used Eq. (2b) to perform a correlation analysis, the following correlation equation was obtained:

(8) $$\begin{array}{c} \log{\text{P}}_{\text{OW}}=0.0206V-8.4281E_{HOMO}-17.0598E_{LUMO}+0.3315\\ R=0.9768,\;R^{2}=0.9541,\;S=0.247,\;n=207,\;F=1406,\;R_{\text{CV}}=0.9760,\;S_{\text{CV}}=0.249 \end{array}$$

**Figure 2.**
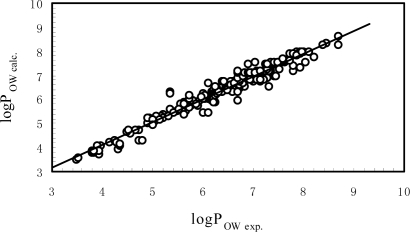
The plot of experimental *n*-octanol/water partition coefficient vs. the ones calculated by Eq. (8).

Figure 2 is the plot of experimental logP_OW_ versus the calculated ones by Eq. (8), which shows that Eq. (8) has a good correlation and prediction ability. The standard deviation of the correlation equation is only 0.247 log units, which is within the experimental uncertainties. The result of Eq. (8) showed that the model (i.e., Eq. (2b)) can be employed to predict logW or logP_OW_ for a wide range compounds with various structures.

It should be noted that some excellent software (such as ACD/LogP, CLogP [28], and so on) have been developed to compute logP_OW_. In order to compare the presented results with the data calculated by these softwares, some leading compounds were selected (see Table 4) and their logP_OW_ were computed by Eq. (8) and by CLogP software (using CLogP packages in SYBYL 6.7 of Tripos, Inc.), respectively. For these compounds in Table 4, the average absolute deviation is 0.45 log units between the experimental logP_OW exp._ and the logP_OW CLogP_ calculated by CLogP software. While, for the same compounds in Table 4, the average absolute deviation is only 0.14 log units between the logP_OW exp._ and the logP_OW calc._ predicted by Eq. (8). Seen from the average absolute deviation, the precision of present method is a little better than that of CLogP software.

## 5. Conclusions

Based on the comprehension of the dissolution process, a very simple three-parameter model was proposed to predict the aqueous solubility and *n*-octanol/water partition coefficients for organohalogen compounds containing nonhydrogen-binding interactions. The model has satisfactory prediction accuracy. Furthermore, every item in the model has a very explicit meaning, which would be helpful to understand the structure-solubility relationships.

## Figures and Tables

**Table 1.** The aqueous solubility (logW) and *n*-octanol/water partition coefficient (logP_OW_) of some organohalogen compounds.

*Substitution patterns*	*V*	*E*_*HOMO*_*(a.u.)*	*E*_*LUMO*_*(a.u.)*	*-logW*_*exp.*_*a*	*logP*_*OW exp.*_*b*	*-logW*_*calc.*_*c*	*logP*_*OW calc.*_*d*	*-ΔlogW**e*	*ΔlogP*_*OW*_*e*
Chlorobiphenyls									
2-	172.9	−0.31684	0.11773		4.38	4.82	4.55		−0.17
3-	172.9	−0.31044	0.10490	5.39	4.66	5.15	4.71	0.24	−0.05
4-	172.9	−0.30610	0.10507	5.33	4.63	5.08	4.67	0.25	−0.04
2,2’-	185.8	−0.33398	0.12177	5.72	4.72	5.31	4.89	0.41	−0.17
2,3-	185.8	−0.32596	0.10991	5.35	4.99	5.58	5.02	−0.23	−0.03
2,3’-	185.8	−0.32760	0.10802	5.26	4.84	5.66	5.07	−0.40	−0.23
2,4-	185.8	−0.32248	0.10594	5.56	5.15	5.66	5.06	−0.10	0.09
2,4’-	185.8	−0.32189	0.10697	5.46	5.09	5.62	5.04	−0.16	0.05
2,5-	185.8	−0.32464	0.10552		5.00	5.70	5.09		−0.09
2,6-	185.8	−0.33322	0.11642		4.93	5.47	4.97		−0.04
3,3’-	185.8	−0.32076	0.09420	6.45	5.27	6.02	5.25	0.43	0.02
3,4-	185.8	−0.31429	0.09528	6.39	5.23	5.89	5.17	0.50	0.06
3,4’-	185.8	−0.31601	0.09419	6.40	5.15	5.95	5.21	0.45	−0.06
3,5-	185.8	−0.32035	0.09364		5.40	6.03	5.25		0.15
4,4’-	185.8	−0.31190	0.09439	6.37	5.23	5.88	5.17	0.49	0.06
2,2’,3-	198.7	−0.33898	0.11209	6.10	5.12	6.07	5.36	0.03	−0.24
2,2’,4-	198.7	−0.34012	0.11041	6.49	5.39	6.14	5.40	0.35	−0.01
2,2’,5-	198.7	−0.33646	0.11028	6.17	5.33	6.09	5.37	0.08	−0.04
2,2’,6-	198.7	−0.33558	0.11438	5.90	5.04	5.94	5.29	−0.04	−0.25
2,3,3’-	198.7	−0.33701	0.10200		5.60	6.37	5.52		0.08
2,3,4-	198.7	−0.32989	0.09988	6.18	5.68	6.33	5.49	−0.15	0.19
2,3,4’-	198.7	−0.33020	0.10032	5.80	5.29	6.32	5.49	−0.52	−0.20
2,3,6-	198.7	−0.33795	0.10464	6.49	5.44	6.29	5.48	0.20	−0.04
2,3’,4-	198.7	−0.33268	0.09730	6.11	5.54	6.46	5.56	−0.35	−0.02
2,3’,5-	198.7	−0.33037	0.09875	6.14	5.65	6.38	5.52	−0.24	0.13
2,4,4’-	198.7	−0.33669	0.09729	6.22	5.71	6.52	5.59	−0.30	0.12
2,4,5-	198.7	−0.33641	0.10953		5.74	6.11	5.38		0.36
2,4,6-	198.7	−0.32716	0.09635		5.50	6.41	5.53		−0.03
2,4’,5-	198.7	−0.32866	0.09554	6.18	5.68	6.46	5.56	−0.28	0.12
2,4’,6-	198.7	−0.33949	0.10293	6.16	5.24	6.37	5.52	−0.21	−0.28
2,3’,4’-	198.7	−0.32913	0.09591	6.21	5.71	6.45	5.55	−0.24	0.16
2,3’,5’-	198.7	−0.33555	0.10933	6.30	5.71	6.11	5.38	0.19	0.33
3,3’,5-	198.7	−0.33039	0.08369		5.70	6.87	5.77		−0.07
3,4,4’-	198.7	−0.31950	0.08533		5.22	6.66	5.65		−0.43
2,2’,3,3’-	211.6	−0.34469	0.10442	6.83	5.67	6.77	5.80	0.06	−0.13
2,2’,3,4-	211.6	−0.34347	0.10180	7.00	5.79	6.84	5.84	0.16	−0.05
2,2’,3,4’-	211.6	−0.34602	0.10288	6.96	5.72	6.84	5.84	0.12	−0.12
2,2’,3,5’-	211.6	−0.34073	0.10278	6.91	5.73	6.76	5.80	0.15	−0.07
2,2’,3,6-	211.6	−0.33988	0.10317	6.30	4.84	6.74	5.78	−0.44	−0.94
2,2’,3,6’-	211.6	−0.34164	0.10791	6.30	4.84	6.61	5.72	−0.31	−0.88
2,2’,4,4’-	211.6	−0.34756	0.10135	7.23	5.94	6.91	5.88	0.32	0.06
2,2’,4,5-	211.6	−0.34328	0.10017	6.86	5.69	6.89	5.86	−0.03	−0.17
2,2’,4,5’-	211.6	−0.34181	0.10125	7.12	5.87	6.83	5.83	0.29	0.04
2,2’,4,6-	211.6	−0.34116	0.10154	6.94	5.75	6.81	5.82	0.13	−0.07
2,2’,4,6’-	211.6	−0.34355	0.10650	6.65	5.51	6.68	5.76	−0.03	−0.25
2,2’,5,5’-	211.6	−0.34136	0.10118	7.00	5.79	6.82	5.83	0.18	−0.04
2,2’,5,6’-	211.6	−0.33758	0.10649	6.65	5.55	6.60	5.71	0.05	−0.16
2,2’,6,6’-	211.6	−0.34062	0.11159	6.20	5.24	6.47	5.65	−0.27	−0.41
2,3,3’,4-	211.6	−0.34043	0.09287	6.77	6.10	7.08	5.97	−0.31	0.13
2,3,4,4’-	211.6	−0.33393	0.09127	6.86	6.24	7.04	5.94	−0.18	0.30
2,3,4,5-	211.6	−0.33570	0.08962		6.05	7.12	5.98		0.07
2,3,4’,5-	211.6	−0.33694	0.08918	6.77	6.10	7.15	6.00	−0.38	0.10
2,3,4’,6-	211.6	−0.33980	0.09821	7.02	5.76	6.90	5.87	0.12	−0.11
2,3,5,6-	211.6	−0.34219	0.09374	7.25	5.96	7.08	5.97	0.17	−0.01
2,3’,4,4’-	211.6	−0.33518	0.08905	6.63	5.98	7.13	5.99	−0.50	−0.01
2,3’,4,5-	211.6	−0.33790	0.08747		6.32	7.22	6.04		0.28
2,3’,4,6-	211.6	−0.34162	0.09677	7.26	6.03	6.97	5.91	0.29	0.12
2,3’,4’,5-	211.6	−0.33665	0.08847	6.69	6.22	7.17	6.01	−0.48	0.21
2,3’,4’,6-	211.6	−0.33738	0.08940	7.02	5.76	7.15	6.00	−0.13	−0.24
2,4,4’,5-	211.6	−0.34486	0.10227	6.77	6.10	6.84	5.84	−0.07	0.26
2,4,4’,6-	211.6	−0.33283	0.08676	7.26	6.03	7.17	6.01	0.09	0.02
2,3’,4’,5’-	211.6	−0.34118	0.09656	6.71	5.98	6.97	5.91	−0.26	0.07
3,3’,4,4’-	211.6	−0.32696	0.07703		6.21	7.40	6.12		0.09
3,3’,5,5’-	211.6	−0.34046	0.07418		6.10	7.70	6.28		−0.18
2,2’,3,3’,6-	224.5	−0.34538	0.09793	6.78	5.60	7.36	6.19	−0.58	−0.59
2,2’,3,4,4’-	224.5	−0.35109	0.09425	7.62	6.18	7.56	6.30	0.06	−0.12
2,2’,3,4,5-	224.5	−0.34736	0.09147	7.87	6.38	7.60	6.31	0.27	0.07
2,2’,3,4,5’-	224.5	−0.34484	0.09419	7.66	6.23	7.47	6.25	0.19	−0.02
2,2’,3,4,6-	224.5	−0.34424	0.09240	7.92	6.50	7.52	6.27	0.40	0.23
2,2’,3,4,6’-	224.5	−0.34923	0.09995	6.78	5.60	7.35	6.18	−0.57	−0.58
2,2’,3,4’,5-	224.5	−0.34906	0.09275	7.82	6.32	7.58	6.31	0.24	0.01
2,2’,3,4’,6-	224.5	−0.34780	0.09405	7.17	5.87	7.52	6.27	−0.35	−0.40
2,2’,3,5,5’-	224.5	−0.34726	0.09667	7.82	6.32	7.43	6.22	0.39	0.10
2,2’,3,5,6-	224.5	−0.34678	0.09634	7.40	6.06	7.43	6.22	−0.03	−0.16
2,2’,3,5’,6-	224.5	−0.34390	0.09255	7.19	5.92	7.51	6.27	−0.32	−0.35
2,2’,3,4’,5’-	224.5	−0.34157	0.09673	7.76	6.30	7.34	6.17	0.42	0.13
2,2’,3,4’,6’-	224.5	−0.34292	0.10112	7.40	6.04	7.22	6.11	0.18	−0.07
2,2’,4,4’,5-	224.5	−0.34864	0.09267	7.95	6.41	7.58	6.30	0.37	0.11
2,2’,4,4’,6-	224.5	−0.34908	0.09510	7.66	6.23	7.50	6.27	0.16	−0.04
2,2’,4,5,5’-	224.5	−0.34567	0.09264		6.65	7.54	6.28		0.37
2,2’,4,5’,6-	224.5	−0.34263	0.09516	7.47	6.11	7.41	6.21	0.06	−0.10
2,3,3’,4,4’-	224.5	−0.34251	0.08561	7.52	6.79	7.72	6.37	−0.20	0.42
2,3,3’,4,5-	224.5	−0.34496	0.08289	7.68	6.92	7.84	6.44	−0.16	0.48
2,3,3’,4’,6-	224.5	−0.34592	0.08472	7.65	6.20	7.80	6.42	−0.15	−0.22
2,3,3’,5,6-	224.5	−0.35128	0.08237	7.95	6.41	7.95	6.50	0.00	−0.09
2,3,3’,5’,6-	224.5	−0.34429	0.08794	7.76	6.45	7.67	6.35	0.09	0.10
2,3,4,4’,5-	224.5	−0.34871	0.09230	7.50	6.71	7.59	6.31	−0.09	0.40
2,3,4,4’,6-	224.5	−0.33919	0.08172	7.96	6.44	7.80	6.41	0.16	0.03
2,3,4’,5,6-	224.5	−0.34518	0.08471	7.88	6.39	7.79	6.41	0.09	−0.02
2,3’,4,4’,5-	224.5	−0.34360	0.08771	7.33	6.57	7.67	6.35	−0.34	0.22
2,3’,4,4’,6-	224.5	−0.34027	0.08002	7.91	6.40	7.87	6.45	0.04	−0.05
2,3’,4,5,5’-	224.5	−0.34174	0.08062		6.30	7.87	6.45		−0.15
2,3’,4,5’,6-	224.5	−0.34715	0.09151	7.92	6.42	7.59	6.31	0.33	0.11
2,3’,4,4’,5’-	224.5	−0.34973	0.09068	7.42	6.64	7.66	6.35	−0.24	0.29
2,2’,3,3’,4,4’-	237.4	−0.35717	0.08854		6.96	8.21	6.71		0.25
2,2’,3,3’,4,5-	237.4	−0.35284	0.08620	8.42	6.76	8.22	6.71	0.20	0.05
2,2’,3,3’,4,5’-	237.4	−0.35195	0.08719		7.30	8.17	6.69		0.61
2,2’,3,3’,4,6-	237.4	−0.34963	0.08771	8.48	6.78	8.12	6.66	0.36	0.12
2,2’,3,3’,4,6’-	237.4	−0.35075	0.09200	7.65	6.20	8.00	6.60	−0.35	−0.40
2,2’,3,3’,5,5’-	237.4	−0.35253	0.08590		6.72	8.22	6.72		0.00
2,2’,3,3’,5,6-	237.4	−0.34916	0.08788	7.65	6.20	8.11	6.65	−0.46	−0.45
2,2’,3,3’,5,6’-	237.4	−0.34856	0.09087	7.82	6.32	8.00	6.60	−0.18	−0.28
2,2’,3,3’,6,6’-	237.4	−0.34611	0.09568		6.96	7.81	6.50		0.46
2,2’,3,4,4’,5-	237.4	−0.35384	0.08494	8.52	6.82	8.27	6.74	0.25	0.08
2,2’,3,4,4’,5’-	237.4	−0.35173	0.08704	8.38	6.73	8.17	6.69	0.21	0.04
2,2’,3,4,4’,6’-	237.4	−0.35560	0.09050	8.24	6.58	8.12	6.66	0.12	−0.08
2,2’,3,4,5,5’-	237.4	−0.34844	0.08494	8.42	6.75	8.20	6.70	0.22	0.05
2,2’,3,4,5,6’-	237.4	−0.35021	0.09052	8.13	6.56	8.04	6.62	0.09	−0.06
2,2’,3,4,5’,6-	237.4	−0.34545	0.08651	8.01	6.45	8.10	6.65	−0.09	−0.20
2,2’,3,4’,5,5’-	237.4	−0.35230	0.08574	8.58	6.85	8.23	6.72	0.35	0.13
2,2’,3,4’,5’,6-	237.4	−0.34866	0.09070	7.94	6.41	8.01	6.60	−0.07	−0.19
2,2’,3,5,5’,6-	237.4	−0.34523	0.08657	7.93	6.42	8.09	6.64	−0.16	−0.22
2,2’,4,4’,5,5’-	237.4	−0.35172	0.08561	8.49	6.80	8.22	6.71	0.27	0.09
2,2’,4,4’,5,6’-	237.4	−0.34981	0.08924	8.12	6.65	8.07	6.64	0.05	0.01
2,2’,4,4’,6,6’-	237.4	−0.35885	0.09295	8.12	6.54	8.09	6.65	0.03	−0.11
2,3,3’,4,4’,5-	237.4	−0.34708	0.07635	8.31	7.44	8.46	6.83	−0.15	0.61
2,3,3’,4,4’,6-	237.4	−0.34978	0.08301	8.48	6.78	8.28	6.74	0.20	0.04
2,3,3’,4’,5,6-	237.4	−0.34945	0.08314	8.48	6.78	8.27	6.74	0.21	0.04
2,3,3’,4’,5’,6-	237.4	−0.35415	0.08765	8.27	6.63	8.19	6.70	0.08	−0.07
2,3,3’,5,5’,6-	237.4	−0.35226	0.08243		7.00	8.33	6.77		0.23
2,3’,4,4’,5,5’-	237.4	−0.34670	0.07271	8.21	7.29	8.57	6.89	−0.36	0.40
3,3’,4,4’,5,5’-	237.4	−0.34085	0.06119	8.85	7.55	8.86	7.04	−0.01	0.51
2,2’,3,3’,4,4’,5-	250.3	−0.35707	0.08042	8.90	7.08	8.84	7.11	0.06	−0.03
2,2’,3,3’,4,4’,6-	250.3	−0.35814	0.08285		7.11	8.77	7.08		0.03
2,2’,3,3’,4,5,5’-	250.3	−0.35537	0.07930	9.10	7.21	8.85	7.12	0.25	0.09
2,2’,3,3’,4,5,6’-	250.3	−0.35338	0.08477	8.59	6.85	8.64	7.01	−0.05	−0.16
2,2’,3,3’,4,5’,6-	250.3	−0.35238	0.08192	8.68	6.92	8.72	7.05	−0.04	−0.13
2,2’,3,3’,4,6,6’-	250.3	−0.35462	0.08300	8.15	6.55	8.72	7.05	−0.57	−0.50
2,2’,3,3’,4,5’,6’	250.3	−0.35079	0.08562	8.42	6.73	8.57	6.97	−0.15	−0.24
2,2’,3,3’,5,5’,6-	250.3	−0.35207	0.08211	8.59	6.85	8.71	7.04	−0.12	−0.19
2,2’,3,3’,5,6,6’-	250.3	−0.34986	0.08556	7.94	6.41	8.56	6.96	−0.62	−0.55
2,2’,3,4,4’,5,5’-	250.3	−0.35515	0.07911	9.10	7.21	8.85	7.12	0.25	0.09
2,2’,3,4,4’,5,6-	250.3	−0.35418	0.07794	8.97	7.13	8.88	7.13	0.09	0.00
2,2’,3,4,4’,5,6’-	250.3	−0.35507	0.08347	8.68	6.92	8.71	7.04	−0.03	−0.12
2,2’,3,4,4’,5’,6-	250.3	−0.35243	0.08170	8.85	7.04	8.73	7.05	0.12	−0.01
2,2’,3,4,5,5’,6-	250.3	−0.34792	0.07777	8.75	6.99	8.79	7.08	−0.04	−0.09
2,2’,3,4’,5,6,6’-	250.3	−0.35225	0.08459	8.49	6.78	8.63	7.00	−0.14	−0.22
2,3,3’,4,4’,5,5’-	250.3	−0.35350	0.06968	8.72	7.72	9.14	7.27	−0.42	0.45
2,3,3’,4,4’,5,6-	250.3	−0.35197	0.07472	8.91	7.08	8.95	7.17	−0.04	−0.09
2,3,3’,4,4’,5’,6-	250.3	−0.35930	0.07838	9.10	7.21	8.94	7.17	0.16	0.04
2,3,3’,4,5,5’,6-	250.3	−0.35485	0.07407	9.10	7.21	9.01	7.20	0.09	0.01
2,3,3’,4’,5,5’,6-	250.3	−0.35800	0.07853	9.10	7.21	8.91	7.15	0.19	0.06
2,2’,3,3’,4,4’,5,5’-	263.2	−0.36003	0.07389	9.70	7.62	9.46	7.51	0.24	0.11
2,2’,3,3’,4,4’,5,6-	263.2	−0.36014	0.07477	9.29	7.35	9.43	7.50	−0.14	−0.15
2,2’,3,3’,4,4’,5,6’-	263.2	−0.35760	0.07749	9.42	7.43	9.31	7.43	0.11	0.00
2,2’,3,3’,4,4’,6,6’-	263.2	−0.35828	0.08100	9.10	7.21	9.20	7.38	−0.10	−0.17
2,2’,3,3’,4,5,5’,6-	263.2	−0.35470	0.07375	9.42	7.43	9.39	7.47	0.03	−0.04
2,2’,3,3’,4,5,5’,6’-	263.2	−0.35688	0.07765	9.10	7.21	9.29	7.42	−0.19	−0.21
2,2’,3,3’,4,5,6,6’-	263.2	−0.35309	0.07686	9.20	7.30	9.26	7.41	−0.06	−0.11
OctaCl-									
2,2’,3,3’,4,5’,6,6’-	263.2	−0.35469	0.08120	9.29	7.35	9.14	7.35	0.15	0.00
2,2’,3,4,4’,5,5’,6-	263.2	−0.35469	0.07336	9.50	7.49	9.40	7.48	0.10	0.01
2,2’,3,4,4’,5,6,6’-	263.2	−0.35822	0.07589	9.48	7.48	9.37	7.47	0.11	0.01
2,3,3’,4,4’,5,5’,6-	263.2	−0.36143	0.07507	9.70	7.62	9.44	7.51	0.26	0.11
2,2’,3,3’,4,4’,5,5’,6-	276.1	−0.35975	0.07059	10.18	7.94	9.93	7.83	0.25	0.11
2,2’,3,3’,4,4’,5,6,6’-	276.1	−0.36005	0.07278	10.07	7.88	9.87	7.80	0.20	0.08
2,2’,3,3’,4,5,5’,6,6’-	276.1	−0.35684	0.07291		8.20	9.81	7.77		0.43
DecaCl-	289.0	−0.36182	0.07013		8.20	10.35	8.12		0.08
Chloronaphthalenes									
1-	143.1	−0.29516	0.08950		4.24		4.23		0.01
2-	143.1	−0.29795	0.09062		4.14		4.24		−0.10
1,2-	156.0	−0.30423	0.07999		4.42		4.74		−0.32
1,4-	156.0	−0.30258	0.07629		4.66		4.79		−0.13
1,5-	156.0	−0.30286	0.07636		4.67		4.79		−0.12
1,7-	156.0	−0.30507	0.07757		4.56		4.79		−0.23
1,8-	156.0	−0.29812	0.07750		4.41		4.73		−0.32
2,3-	156.0	−0.30553	0.07967		4.71		4.75		−0.04
2,7-	156.0	−0.30912	0.07857		4.81		4.80		0.01
1,3,7-	168.9	−0.31340	0.06630		5.35		5.31		0.04
2,3,6-	168.9	−0.31512	0.06818		5.12		5.30		−0.18
1,2,3,4-	181.8	−0.31569	0.05984		5.75		5.71		0.04
1,2,3,5-	181.8	−0.31696	0.05772		5.77		5.75		0.02
1,3,5,7-	181.8	−0.31919	0.05479		6.19		5.82		0.37
1,3,5,8-	181.8	−0.31355	0.05442		5.76		5.78		−0.02
1,4,6,7-	181.8	−0.31813	0.05561		5.81		5.80		0.01
Chlorobenzenes									
Mono-	102.2	−0.33466	0.13195		2.98		3.00		−0.02
1,2-	113.1	−0.34231	0.11787		3.38		3.53		−0.15
1,3-	114.6	−0.34436	0.11598		3.48		3.61		−0.13
1,4-	115.2	−0.33830	0.11560		3.38		3.58		−0.20
1,2,3-	125.3	−0.35227	0.10497		4.04		4.08		−0.04
1,2,4-	128.1	−0.34649	0.10290		3.98		4.13		−0.15
1,3,5-	128.1	−0.35843	0.10131		4.02		4.26		−0.24
1,2,3,4-	141.0	−0.35244	0.09274		4.55		4.62		−0.07
1,2,3,5-	141.0	−0.35558	0.09100		4.65		4.67		−0.02
1,2,4,5-	141.0	−0.35147	0.09104		4.51		4.64		−0.13
Penta-	153.9	−0.35809	0.08139		5.03		5.12		−0.09
Hexa-	166.8	−0.36423	0.07241		5.47		5.59		−0.12
3,4-Dimethyl-	131.6	−0.31901	0.13266		3.82		3.46		0.36
Chlorotoluenes									
2-	116.9	−0.32902	0.13290		3.42		3.24		0.18
3-	118.1	−0.32981	0.13103		3.28		3.30		−0.02
4-	118.3	−0.32396	0.13090		3.33		3.26		0.07
2,4-	129.0	−0.33516	0.11725		4.24		3.81		0.43
2,6-	126.9	−0.34055	0.11804		4.29		3.80		0.49
2,3-diCl-*p*-cymene	190.0	−0.32996	0.12206		5.50		4.94		0.56
2,5-diCl-*p*-cymene	190.0	−0.32714	0.11994		5.60		4.95		0.65
2,3,6-triCl-*p*-cymene	202.9	−0.33672	0.10871		6.20		5.49		0.71
TetraCl-*p*-cymene	215.8	−0.34333	0.09615		6.80		6.02		0.78
Bromobenzenes									
Mono	105.5	−0.33082	0.12952		3.02		3.08		−0.06
1,2-	121.6	−0.33684	0.11453		3.64		3.72		−0.08
1,3-	121.6	−0.33898	0.11280		3.75		3.76		−0.01
1,4-	121.6	−0.33232	0.11143		3.79		3.73		0.06
1,3,5-	137.7	−0.35159	0.09877		4.51		4.44		0.07
1,2,4,5-	153.8	−0.34276	0.08639		5.13		4.91		0.22
Hexa-	186.0	−0.35176	0.06184		5.73		6.06		−0.33
Bromotoluenes									
2-	120.2	−0.32632	0.13018		3.42		3.33		0.09
3-	121.3	−0.32684	0.12998		3.28		3.36		−0.08
4-	122.5	−0.32096	0.12962		3.33		3.34		−0.01
Bromochlorobenzenes									
2-	116.8	−0.33903	0.11593		3.83		3.61		0.22
4-	116.8	−0.33494	0.11346		3.83		3.62		0.21

(a) Taken from Ref. [11];

(b) Taken from Ref. [10] and Ref. [23];

(c) Calculated by Eq. (3);

(d) Calculated by Eq. (8);

(e) ΔlogW=logW_exp._ − logW_calc._; ΔlogP_OW_= logP_OW exp._ − logP_OW calc._

**Table 2.** The characteristics of descriptors in Eq. (3)

Descriptor	*V*	*E*_*HOMO*_	*E*_*LUMO*_
***S***	0.0033	3.2308	4.2833
***t*-score**	9.0395	−3.4612	−7.6414
**Significance**	0.0000	0.0007	0.0000

**Table 3.** Interrelations of descriptors in Eq. (3)

	*V*	*E*_*HOMO*_	*E*_*LUMO*_
***V***	1		
***E*_*HOMO*_**	−0.5981	1	
*E*_*LUMO*_	−0.5704	0.2004	1

**Table 4.** The results of logP_OW_ calculation by the presented method and the ClogP software for a few leading organohalogen compounds.

Substitution patterns	logP_OW exp._*a*	logP_OW calc._*b*	logP_OW CLogP_*c*	ΔlogP_OW_*d*	ΔlogP_OW_*e*
Chlorobiphenyls					
2-	4.38	4.55	4.49	−0.17	−0.11
4-	4.63	4.67	4.74	−0.04	−0.11
2,2’-	4.72	4.89	4.96	−0.17	−0.24
2,3-	4.99	5.02	5.09	−0.03	−0.10
2,6-	4.93	4.97	4.96	−0.04	−0.03
4,4’-	5.23	5.17	5.46	0.06	−0.23
2,2’,4-	5.39	5.40	5.67	−0.01	−0.28
2,2’,5-	5.33	5.37	5.67	−0.04	−0.34
2,3,4-	5.68	5.49	5.68	0.19	0.00
2,3,4’-	5.29	5.49	5.80	−0.20	−0.51
2,4,6-	5.50	5.53	5.67	−0.03	−0.17
2,2’,3,3’-	5.67	5.80	6.14	−0.13	−0.47
2,2’,3,5’-	5.73	5.80	6.26	−0.07	−0.53
2,2’,4,4’-	5.94	5.88	6.38	0.06	−0.44
2,3,4,5-	6.05	5.98	6.39	0.07	−0.34
2,3,5,6-	5.96	5.97	6.26	−0.01	−0.30
3,3’,4,4’-	6.21	6.12	6.64	0.09	−0.43
2,2’,3,3’,6-	5.60	6.19	6.60	−0.59	−1.00
2,2’,3,4,4’-	6.18	6.30	6.85	−0.12	−0.67
2,2’,3,5,5’-	6.32	6.22	6.97	0.10	−0.65
2,3,4,4’,5-	6.71	6.31	7.10	0.40	−0.39
2,3,4,4’,6-	6.44	6.41	6.97	0.03	−0.53
2,2’,3,4,4’,5-	6.82	6.74	7.57	0.08	−0.75
2,3,3’,4,4’,5-	7.44	6.83	7.70	0.61	−0.26
2,3,3’,4,4’,6-	6.78	6.74	7.57	0.04	−0.79
2,2’,3,3’,4,5,5’-	7.21	7.12	8.16	0.09	−0.95
2,2’,3,4,4’,5,6-	7.13	7.13	8.15	0.00	−1.02
2,2’,3,4,5,5’,6-	6.99	7.08	8.15	−0.09	−1.16
2,3,3’,4,4’,5,5’-	7.72	7.27	8.29	0.45	−0.57
2,2’,3,3’,4,4’,5,5’-	7.62	7.51	8.75	0.11	−1.13
2,2’,3,3’,4,4’,5,6-	7.35	7.50	8.62	−0.15	−1.27
2,2’,3,3’,4,5’,6,6’-	7.35	7.35	8.49	0.00	−1.14
2,2’,3,3’,4,4’,5,5’,6-	7.94	7.83	9.34	0.11	−1.40
2,2’,3,3’,4,4’,5,6,6’-	7.88	7.80	9.21	0.08	−1.33
Deca-	8.20	8.12	9.92	0.08	−1.72
Chloronapthalenes					
2-	4.14	4.24	4.03	−0.10	0.11
1,4-	4.66	4.79	4.74	−0.13	−0.08
1,7-	4.56	4.79	4.74	−0.23	−0.18
2,3-	4.71	4.75	4.62	−0.04	0.09
2,3,6-	5.12	5.30	5.34	−0.18	−0.22
1,2,3,5-	5.77	5.75	5.93	0.02	−0.16
1,4,6,7-	5.81	5.80	6.05	0.01	−0.24
Chlorobenzenes					
1,2-	3.38	3.53	3.45	−0.15	−0.07
1,2,3-	4.04	4.08	4.04	−0.04	0.00
1,3,5-	4.02	4.26	4.28	−0.24	−0.26
1,2,3,4-	4.55	4.62	4.63	−0.07	−0.08
Penta-	5.03	5.12	5.35	−0.09	−0.32
Hexa-	5.47	5.59	6.06	−0.12	−0.59
3,4-Dimethyl-	3.82	3.46	3.80	0.36	0.02
Chlorotoluenes					
2-	3.42	3.24	3.35	0.18	0.07
2,6-	4.29	3.80	4.07	0.49	0.22
2,5-diCl-*p*-cymene	5.60	4.95	5.49	0.65	0.11
Bromobenzenes					
Mono-	3.02	3.08	3.01	−0.06	0.01
1,3-	3.75	3.76	3.87	−0.01	−0.12
Hexa-	5.73	6.06	6.72	−0.33	−0.99
4-Cl-	3.83	3.62	3.72	0.21	0.11
Bromotoluenes					
4-	3.33	3.34	3.50	−0.01	−0.17

(a) Taken from Ref. [10] and Ref. [23];

(b) Calculated by Eq. (8);

(c) Calculated by CLogP software;

(d) logW=logW_exp._ − logW_calc._;

(e) ΔlogP_OW_=logP_OW exp._ − logP_OW CLogP_
